# Brodie's Abscess of the Thumb: A Rare Complication of a Dog Bite

**DOI:** 10.1177/22925503261436340

**Published:** 2026-05-06

**Authors:** Jacob Bouchard, Stefan Padeanu, Ophélie Doucet, Dominique Tremblay

**Affiliations:** 1 Division of Plastic and Reconstructive Surgery, 5622 University of Montreal, Montreal, QC, Canada; 2 Division of Plastic and Reconstructive Surgery, University of Montreal, 5622 Maisonneuve-Rosemont Hospital, Montreal, QC, Canada

**Keywords:** abscess, hand, bone infection, osteomyelitis, animal bite, abcès, infection de l’os, main, morsure d’animal, ostéomyélite

## Abstract

Brodie's abscess is a rare form of subacute osteomyelitis, most commonly affecting the metaphysis of long bones in pediatric patients. Involvement of the hand is exceptional, and its indolent course combined with nonspecific imaging features can make diagnosis challenging. Here, we describe a case of Brodie's abscess following an animal bite and compare it with the few reported cases of Brodie's abscess of the hand in the literature.

## Résumé

L’abcès de Brodie est une forme rare d’ostéomyélite subaiguë, qui touche surtout la métaphyse des os longs chez les patients d’âge pédiatrique. Il est exceptionnel qu’il touche la main, et son évolution indolente combinée à ses caractéristiques non spécifiques à l’imagerie peut en compliquer le diagnostic. Les auteurs décrivent un cas d’abcès de Brodie après une morsure d’animal et le comparent aux quelques cas déclarés d’abcès de Brodie de la main relevés dans les publications scientifiques.

## Introduction

Brodie's abscess, first described by Sir Benjamin Brodie in 1832, is a form of subacute osteomyelitis. It is characterized by a localized, well-circumscribed intramedullary collection of pus surrounded by a cuff of sclerotic bone.^1,2^ The condition is thought to arise from hematogenous or direct seeding of bacteria, often *Staphylococcus aureus.*^3^ Following a period of dormancy, patients tend to present with localized symptoms: tenderness, swelling, and erythema. Systemic symptoms are often absent.^1,3^

Brodie's abscesses can pose significant diagnostic challenges through their insidious onset, vague clinical presentation, and unspecific radiologic characteristics. Most cases recorded in the literature involve the metaphysis of weight-bearing bones of the lower extremity. Brodie's abscesses of the upper extremity are quite rare, particularly at the level of the hand, where, to our knowledge, only 3 cases have been reported in the literature.^3,4^ This case report highlights a rare instance of Brodie's abscess affecting the proximal phalanx of the thumb several weeks after a dog bite.

## Case Report

A healthy, 32-year-old right-hand dominant male presented to the emergency department with a swollen and painful left thumb. His symptoms started 2 weeks prior, having suffered a domestic dog bite approximately 6 weeks earlier. The patient was first seen in an outpatient clinic by a general practitioner following the initial trauma. He was treated conservatively without antibiotics. No systemic symptoms were reported since the injury.

**Figure 1.**
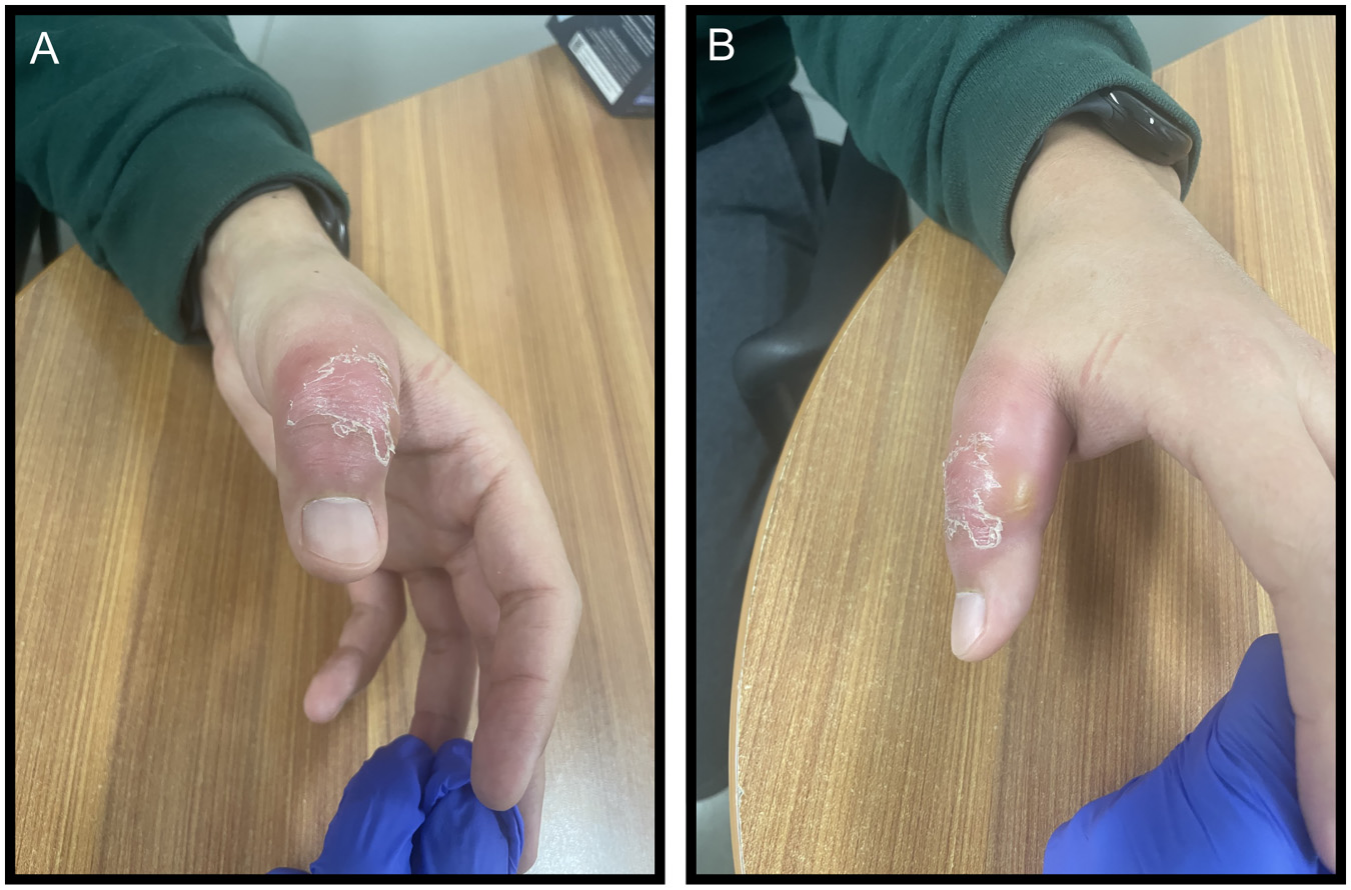
Antero-posterior (A) and oblique (B) views of the left thumb showing localized erythema, edema, and subcutaneous pus.

On physical examination, the patient presented with significant edema and erythema, localized proximally to the interphalangeal joint of the left thumb. A fluctuant mass with visible subcutaneous pus was visible at the dorso-ulnar aspect of the proximal phalanx. No deformities or signs of previous trauma were seen. Palpation was painful over the interphalangeal joint and metacarpophalangeal joint (Figure 1). Diagnostic maneuvers for septic arthritis were negative. Active and passive range of motion were normal and did not elicit increased pain.

**Figure 2.**
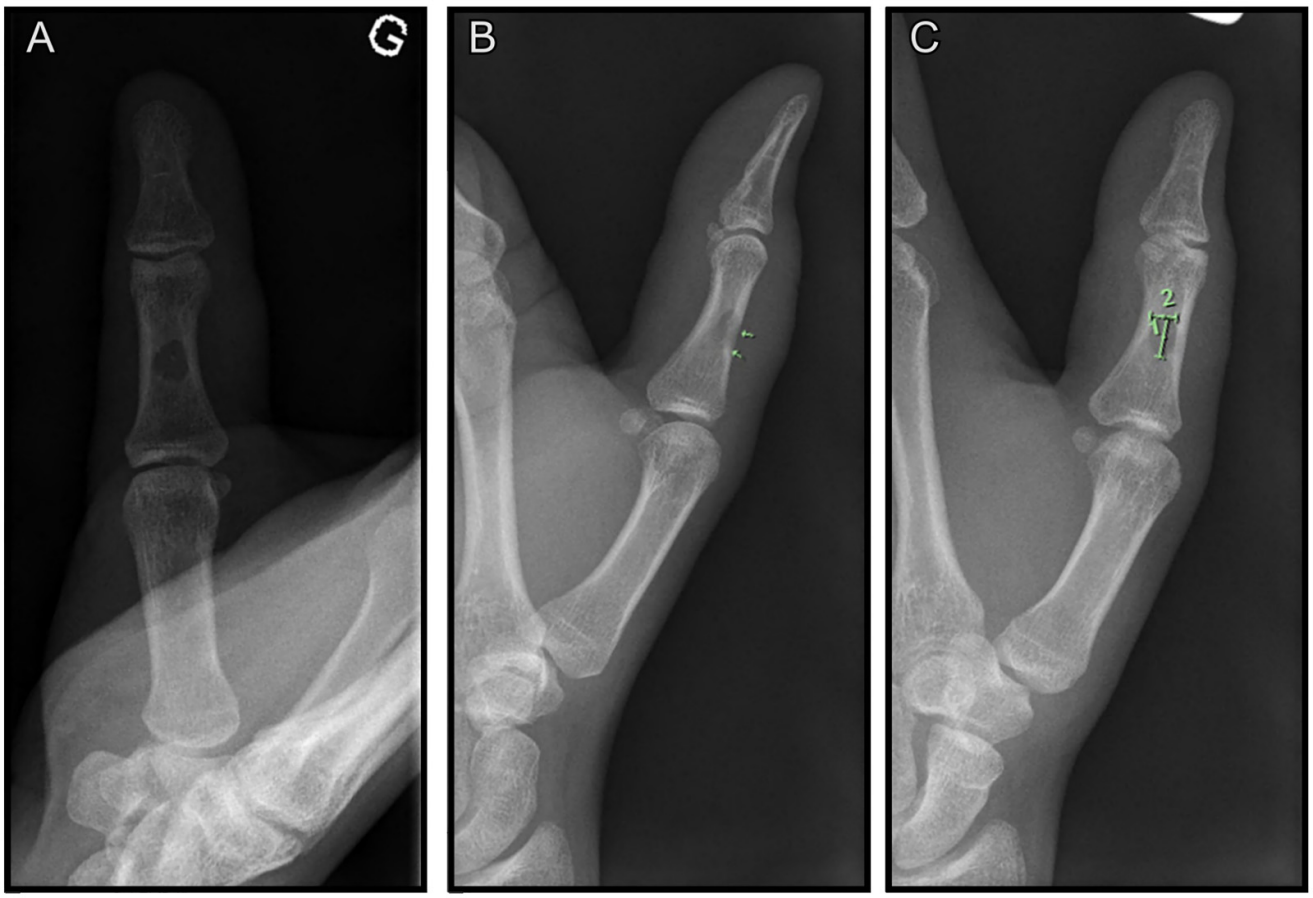
X-ray of the left thumb: antero-posterior view (A), lateral view (B), and oblique view (C); showing a lytic lesion of the mid proximal phalanx. Green annotations show its dimension of 0.68 cm [1] × 0.45 cm [2].

**Figure 3.**
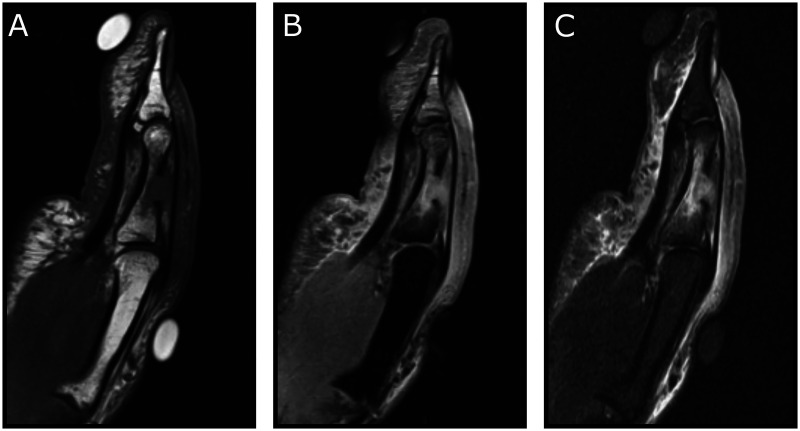
Lateral T1-weighted (A), T2-weighted (B), and STIR (C) sequences of the left thumb MRI showing cortical permeation, in continuity with a subcutaneous panniculitis and phlegmonous changes.

Blood work was normal (WBC 9.2 × 10^9^ cells/L, CRP 4.1 mg/L). X-ray of the left first digit showed a spherical, lytic lesion involving the mid-diaphysis of the proximal phalanx (Figure 2). MRI of the left hand revealed a 7.1 mm by 4 mm lesion with cortical permeation, in continuity with a subcutaneous panniculitis and phlegmonous changes. On Turbo STIR sequences, hyperintensity was observed in the diaphyseal bone marrow. Post-contrast imaging revealed significant enhancement of the proximal phalanx (Figure 3).

**Figure 4.**
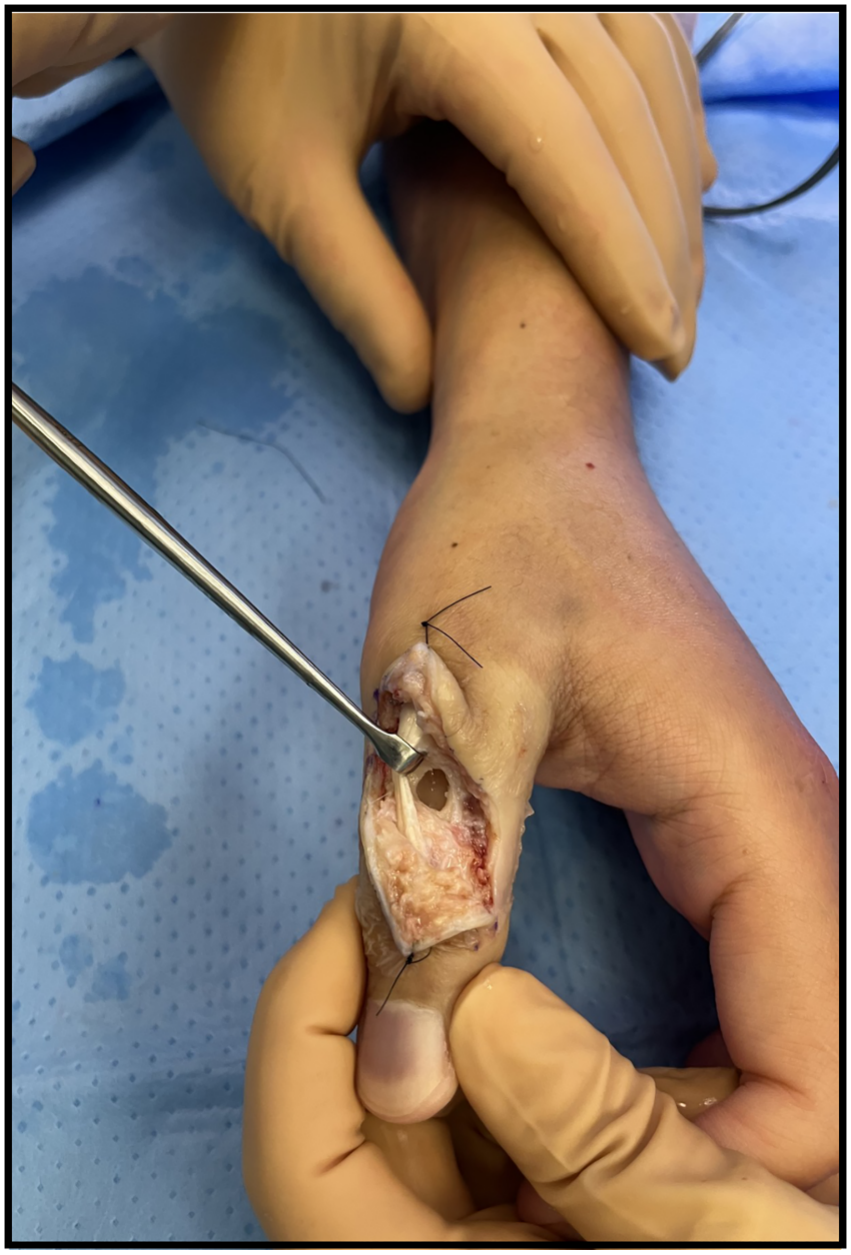
Intraoperative photo showing the intraosseous cavity following surgical debridement of the abscess.

At the time of consultation, the patient received 2 grams of intravenous ceftriaxone. The decision was made to drain the subcutaneous abscess at bedside to limit soft tissue damage. Purulent fluid was sent for bacterial cultures. The patient was then discharged with close follow-up and intravenous antibiotics until definitive surgical irrigation and debridement were performed 4 days later in the operating room (Figure 4). Initial and intraoperative cultures were negative. Given the high suspicion of a bacterial etiology, the patient was treated postoperatively with 2 weeks of intravenous ceftriaxone followed by 4 weeks of oral amoxicillin-clavulanic acid.

**Figure 5.**
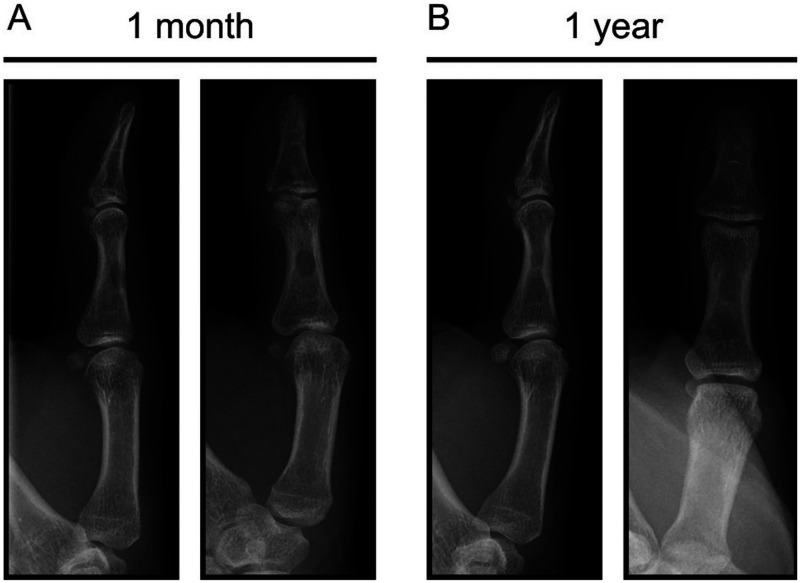
X-ray of the left thumb at 1 month (A) and 1 year (B) post-surgery.

One-month post-treatment, the patient reported no pain and showed no signs of infection recurrence. Physical examination revealed a slight reduction in proximal interphalangeal joint flexion (0°-50°) with full extension. Radiographic evaluation demonstrated stability of the lytic lesion (Figure 5A). At the 1-year follow-up, the patient had returned to his premorbid functional status, exhibiting a full range of motion and resumption of normal activities. X-rays confirmed complete consolidation of the lytic lesion (Figure 5B).

## Discussion

We present one of a few reported cases of a Brodie's abscess of the hand, following a dog bite.^3,4^ Infection following an animal bite is frequent, especially in the hand; however, it rarely develops into a chronic infection.^5–7^ The 8-week interval between the presumed bacterial inoculation from the dog bite and the onset of symptoms suggests a subacute process. Following bony insemination, a medullary abscess developed, followed by cortical permeation and soft tissue invasion, as seen on MRI.

Three other cases of Brodie's abscesses in the hand have been described, two of which are included in a case series for which no detailed history or management was described.^8^ The other case involved a fight bite injury at the metacarpal head sustained several years prior, which appeared to have been reactivated following a blunt trauma, resulting in a cortical break and subsequent acute infection. Physical and paraclinical findings were similar to our patient, with the notable exception of the subcutaneous pus. The authors followed a comparable management approach, beginning with surgical debridement followed by an antibiotic regimen.

As expected, bone erosion due to infection can result in a deficit after abscess evacuation. Typically, large cavities are filled using either autologous bone graft or a substitute.^8^ However, bone grafting is not always necessary in the upper extremities, as these are generally areas of low mechanical load.^9^ In our case, we considered bone grafting unnecessary due to the diaphyseal location of the abscess, the preserved integrity of the cortex in its majority, and the patient's low occupational demands on the bone.

Radiographically, a lytic lesion with a sclerotic border is the hallmark of a Brodie's abscess. However, its presentation may often mimic other bone lesions, making the diagnosis challenging. Additional imaging modalities such as CT and MRI can be useful, as in our case.^10,11^ Biopsy and culture remain the gold standard for a definitive diagnosis, especially to differentiate it from osteoid osteomas, which closely resemble it on imaging.^12^ Unfortunately, in our case, bacterial cultures were negative. We hypothesize that the prior antibiotic administration contributed to the negative bacterial cultures. Nonetheless, a systematic review by van der Naald et al showed no bacterial growth in more than 25% of cases.^3^

## Conclusion

Here we present one of the few reported cases of a Brodie's abscess of the hand, and the first documented following a dog bite. Suspicion of subacute osteomyelitis, which often has an insidious onset and can be easily overlooked, should be raised in patients with a history of potential inoculation risk, such as human or animal bites and/or prior bony fixation. Treatment should be prompt and include a combination of medical and surgical intervention to decrease the chance of long-term disability and optimize outcomes.
